# Cyber-Attacks Risk Analysis Method for Different Levels of Automation of Mining Processes in Mines Based on Fuzzy Theory Use

**DOI:** 10.3390/s20247210

**Published:** 2020-12-16

**Authors:** Agnieszka A. Tubis, Sylwia Werbińska-Wojciechowska, Mateusz Góralczyk, Adam Wróblewski, Bartłomiej Ziętek

**Affiliations:** 1 Faculty of Mechanical Engineering, Wroclaw University of Science and Technology, Wyspianskiego 27, 50-370 Wroclaw, Poland; agnieszka.tubis@pwr.edu.pl; 2 Faculty of GeoEngineering Mining and Geology, Wroclaw University of Science and Technology, Wyspianskiego 27, 50-370 Wroclaw, Poland; mateusz.goralczyk@pwr.edu.pl (M.G.); adam.wroblewski@pwr.edu.pl (A.W.); bartlomiej.zietek@pwr.edu.pl (B.Z.)

**Keywords:** cybersecurity, risk analysis, fuzzy theory, Factory 4.0, mining sector, mining machinery, mines automation level

## Abstract

The rising automation level and development of the Industry 4.0 concept in the mining sector increase the risk of cyber-attacks. As a result, this article focuses on developing a risk analysis method that integrates Kaplan’s and Garrick’s approach and fuzzy theory. The proposed approach takes into account the level of automation of the operating mining processes. Moreover, it follows five main steps, including identifying the automation level in a selected mine, definition of cyber-attack targets, identification of cyber-attack techniques, definition of cyber-attack consequences, and risk ratio assessment. The proposed risk assessment procedure was performed according to three cyber-attack targets (databases, internal networks, machinery) and seven selected types of cyber-attack techniques. The fuzzy theory is implemented in risk parameter estimation for cyber-attack scenario occurrence in the mining industry. To illustrate the given method’s applicability, seven scenarios for three levels of mine automation are analyzed. The proposed method may be used to reveal the current cybersecurity status of the mine. Moreover, it will be a valuable guide for mines in which automation is planned in the near future.

## 1. Introduction

A literature review of publications on risk analysis and risk assessment in mining [1] has revealed that the development of risk-based research on mining machinery operation is an important area of high research potential that now needs to be supplemented. An additional analysis of recent years’ publications indicates a research gap in the analysis of risks related to mining machinery’s cyber-safety. This research gap may be due to the fact that mines had limited possibilities to expand Internet Connection Sharing (ICS) underground and use it in their operations until recently. Therefore, the need for such research was limited.

However, for several years, we have been observing continuous technology development in the mining industry. Many technical solutions are being developed that are innovative, among others, due to the demanding operating conditions in the underground mines and the working regime of the equipment and systems implemented there [2]. Additionally, automation of dangerous, repetitive, and labor-intensive tasks is currently a key factor contributing to improving safety in mines. Therefore, in many mines, we can observe development trends consistent with the Industry 4.0 concept, which lead to the development of mines operating under the assumptions of Factory 4.0. According to the report [3], the following factors indicate the need for the automation of mines:

- Mining is a set of operations related to the extraction and subsequent material flow, including blasting, drilling, or hauling. Automation of such processes gives the possibility of increasing the control level in a random and changeable environment, in which mines have to operate. The main focus is on the implementation of decision-making processes according to a defined set of stringent rules.
- Automation can be a solution to the increasingly difficult and costly process of recruiting skilled labor in unattractive mine locations. Through the use of automation, it is possible to remotely operate the equipment and supervise operations in mines. Thanks to this, it is possible to maintain a smaller workforce in the mine and more efficient use of available human resources.
- Automation provides a number of benefits in the area of mine operations. The main ones are to improve the safety of miners and increase the level of control at each stage of production. Moreover, it allows, among other things, reduction in operating costs, reduction in operating deviations or introduction of monitoring of the implemented operating processes.

Industry 4.0 is a combination of information technology and highly controllable computer-driven machines [4,5]. The Industry 4.0 vision has promoted smart mine concepts in the mining area by augmenting all assets with sensor-based connectivity. However, the development of the Industry 4.0 concept in the mining sector increases the risk of cyber-attacks. These attacks may target automated equipment, automated decision support systems, but also major mining processes as well as logistical support and transportation processes. Cyber-attacks may result in financial losses (caused, for example, by stoppages in the mining process), but above all, they may pose a threat to the safety of miners and even their health or life. Unfortunately, not all decision-makers responsible for mining processes are aware of the severe threat that cyber-attacks currently pose to their sector. According to the report prepared by PricewaterhouseCoopers [6], only 12% of Chief Executive Officers CEOs of mining companies are concerned about cyber threats to their company’s growth prospects. At the same time, the authors of the report indicate three reasons why managers in the mining sector should start analyzing the risks associated with cyber-attacks [6]:

- The use of automated and connected operational technologies (OP) in mines: hackers can gain access to a mine’s network through an organization, which is connected to an information/ communication network and has a low cybersecurity level.
- Threat to the safety of workers, community, and environment: cyber-attacks on mining systems (e.g., ventilation system, pipeline controls, or gas monitors) may be very dangerous and have severe and life-threatening consequences.
- Attackers’ motivation, which can be extremely diverse.

The significant threats posed by cyber-attacks are confirmed by subsequent reports prepared by numerous global institutions. In 2017, the World Economic Forum announced that cyber-security violations are among the five most serious threats facing the world today [7]. This threat also applies to the mining sector. According to a study published by Ernst & Young (EY) [8], 54% of mining and metals companies have had a significant cybersecurity incident in the last year (2019). This is one of the reasons why a cybersecurity incident was ranked fourth among the top 10 business risks in the ranking prepared by EY for the years 2019–2020.

Following the introduction, this research’s primary intention is to develop a risk analysis method that integrates Kaplan and Garrick’s approach [9] and fuzzy theory to assess cyber-attacks’ risk in mines depending on the level of automation of performed mining processes. The proposed fuzzy-based risk analysis method was based on the definition of three mine automation levels: modern mine, real-time mine, and intelligent mine. Moreover, it followed five main steps, including identification of the automation level in a selected mine, definition of cyber-attack targets, identification of cyber-attack techniques, definition of cyber-attack consequences, and risk ratio assessment. The proposed risk assessment procedure includes three cyber-attack targets (databases, internal networks, machinery). Seven selected types of cyber-attack techniques were analyzed for the defined targets, e.g., phishing and network spoofing. Thus, this study’s main contribution is the introduction of the risk-based decision-making method that focuses on risks arising from the Industry 4.0 concept in the mining sector, particularly on the risk of cyber-attacks. The proposed solution takes into account the specificity of the mining sector.

This paper is structured as follows. Section 2 presents a comprehensive literature review on cybersecurity risk methods/models and their implementation possibilities in the mining industry. Later, Section 3 provides a methodological background on the fuzzy theory. This gives the possibility of introducing the proposed method for risk analysis of cyber-attacks in the mining industry in Section 4. The implementation possibilities of the developed method are presented in Section 5. Section 6 presents the results and discussion. Finally, Section 7 provides conclusions, limitations of the study, and suggestions for the authors’ future research works.

## 2. Cyber Risk in the Mining Industry—Related Work

There is no one, unified, and widely used definition of risk term [10]. One of the most often cited risk term definitions is given in PN-ISO 31000 standard [11], where risk is defined as the effect of uncertainty on objectives. A summary of the classification of risk definitions is given in [10,12]. Moreover, the review and discussion of risk perspectives are given in [13,14,15,16,17]. 

Simultaneously, in PKN-ISO Guide 73:2012 [18], standard risk assessment is defined as the overall process of risk identification, risk analysis, and risk evaluation. This process may be performed with the use of different assessment methods. A comprehensive literature review on safety and security and risk assessment methods is presented in [19]. Moreover, the authors in [20] review 26 threat analysis techniques by investigating their possibility of implementing software engineering trends (i.e., DevOps, agile development, Internet of Things, and automotive). The general overview of risk analysis methods is given in [21,22]. Moreover, in work [23], an overview of methods and tools that the International Organization of Securities Commissions (IOSCO) and security regulators have developed and implemented to identify and assess new risks is presented.

Mining is a hazardous operation related to considerable environmental, health, and safety risks to miners [1]. For example, inappropriate shift schedules, excessive working hours, increased pollution problems, adverse environments and work conditions, and lack of training can increase miners’ exposure to risk and result in employee fatigue and danger to the miners’ lives [24]. According to the report [8], the mentioned hazardous situations are supplemented with cyber threats.

Cyber risk applies to a broad range of threats and encompasses a wide and varied range of attacker motivations, goals, modes of attack, and ultimate business impacts [25]. An overview of the most exploited vulnerabilities in existing hardware, software, and network layers is presented in [26]. The issues of cybersecurity risk assessment for supervisory control and data acquisition (SCADA) and distributed control systems (DCSs) were widely analyzed in [27,28]. The authors in their works focused on risk assessment methods such as hierarchical holographic modeling (HHM), inoperability input–output modeling (IIM), and the risk filtering, ranking, and management method (RFRM). Moreover, probability risk analysis (PRA) was also presented, which includes methods such as fault tree analysis (FTA), event tree analysis (ETA), and failure mode and effects analysis (FEMA). Later, in 2015, the authors in work [29] focused on a literature review on cybersecurity risk assessment of SCADA systems. They selected and examined twenty-four risk assessment methods developed for or applied in the context of a SCADA system. They also proposed a categorization of cybersecurity risk assessment methods for SCADA systems according to aspects such as application domain, risk management concepts covered, impact measurement, or evaluation and tool support. Moreover, one may also find surveys on sequential cyber-attacks (see [30]). The authors focused on security risks from cyber-attacks subject to sequence dependence, where the occurrence order of hazardous events matters to the system status and influences the possible negative consequences of occurred hazards.

The cybersecurity for critical infrastructures is analyzed in [31,32] or [33]. These papers primarily focused on the negative consequences of cybersecurity threats to the whole system and its operational costs. In work [31], the authors focused on both physical assets and cybersecurity. In [32], the authors compared the unit commitments models to tackle renewable uncertainty in power systems. The authors of [33] focused on information technology (IT) infrastructure and its resilience to cyber threats. Later, the authors in [34] defined three main areas characteristic for cybersecurity studies:

- Technology, i.e., development of technological solutions that are aimed at reducing or identifying threats and attacks;
- Investments in cybersecurity;
- Risk assessment models focused on measuring the risk of cyberattack occurrence.

More cybersecurity information may also be found in works [35,36]. Moreover, the issues of cyber-crime are surveyed in [37].

Cyber risk in the mining industry results mostly from an increased digital transformation and the convergence of information technology (IT) and operational technology (OT), which makes companies more vulnerable to cyber threats [38]. An interesting approach to cyber risk in the mining industry is presented in [39]. In the mentioned report, the authors discussed the possible cyber hazards that may occur in mines and investigate the issues of assessing cybersecurity controls’ maturity in a corporate context and an operational environment. They also define the five essential controls, i.e., awareness training, access control, network security, portable media, and incident response. Simultaneously, the authors in the report [25] analyzed the specific areas of cyber risk exposure for miners and investigated the potential impact on operations. They also present the mining sector’s key risks: e-mail malware, phishing, spam, data breaches, and identity theft. The examples of cyber-attacks on mines are presented in the EY report [40].

## 3. Fuzzy Theory in Mining Safety Problems

In this paper, the fuzzy set theory is implemented to overcome the uncertainty and lack of knowledge in risk parameter estimation for cyber-attack scenario occurrence in the mining industry.

Zadeh introduced a fuzzy set theory in 1965 [41]. According to [42], three main research domains have been indicated for fuzzy set theory implementation: vague phenomenon (vague relations in the modeling of problems), information vagueness, and heuristic algorithms. Thus, one may use linguistic variables when assessing qualitatively or give intervals instead of exact crisp values in evaluating the data quantitatively. Therefore, the fuzzy set theory provides more descriptive studies [43]. What is also important is that decision-makers tend to believe that it is more certain to make interval judgments than fixed-value judgments.

Many scientific articles develop a given theory and present its application possibilities in specific research areas that have been published due to the significant convenience. One of the survey research studies is given by Shapiro and Koissi [44].

The fuzzy theory has also been proven to be useful in solving mining safety problems. For example, in work [45], the authors propose coal dust explosions fuzzy fault tree analysis combined with the Visual Basic (VB) program. The authors also introduce a new weighting method for expert opinions. Later, in work [46], the authors focus on the occurrence of coal burst accidents. The proposed approach is also based on fuzzy theory and fault tree analysis implementation. The proposed solution is dedicated to underground coal mining.

Moreover, the gas and dust explosion risk in coal mines is analyzed in [47], where the fuzzy fault tree approach is also implemented. Additionally, fuzzy fault tree analysis of accidents and incidents in mining processes is analyzed in [43]. The authors focus on the underground loading and conveying process of the mine.

The underground coal mines’ safety risk is analyzed as well, e.g., in [48]. The authors use the fuzzy reasoning approach and Mamdani fuzzy logic model developed in MATLAB’s fuzzy logic toolbox. In another work [49], the classical decision matrix risk assessment method is extended based on a fuzzy logic-based safety evaluation method implementation. The proposed approach is implemented in mechanized underground coal mines in Turkey.

A bowtie analysis and attack tree are introduced in [50]. The proposed integrated approach provides a broad representation of risk scenarios in terms of industrial control systems’ safety and security.

Research works can be found in the literature that integrate fuzzy theory with multi-criteria decision-making approaches. VIKOR (Vlse Kriterijumska Optimizacija Kompromisno Resenje)-based approach for safety assessment in the mining industry is given in [51]. At the same time, the fuzzy Analytic Hierarchy Process (AHP) method is proposed in [52]. In the given paper, the fuzzy reasoning approach evaluates the risk levels associated with the identified hazard factors. Later, the AHP pairwise comparison matrix is developed to obtain priority weights for the hazard factors. The proposed approach is implemented for metalliferous mines. The fuzzy-grey correlation method with the fuzzy Technique for Order of Preference by Similarity to Ideal Solution (TOPSIS) risk assessment model for Pb-ZN mines is given in [53].

The operational risk in polish mining enterprises from a long-term research perspective is analyzed in [54]. The proposed solution is based on holistic fuzzy evaluation to standardize and aggregate the levels of operational risk assessment.

## 4. Fuzzy-Based Risk Analysis Method

For the purposes of the developed cybersecurity risk assessment methodology, the authors have analyzed the existing literature in order to define what kind of factors in risk analysis should be taken into account for the mining sector, what kind of cyberattacks are the most frequently performed, and they have also investigated the published reports about cybercrime in the mining industry (e.g., [3]). Following the conducted research studies, they have found that the motivation of cyber attackers is significant and should be considered when defining attack scenarios or its target, as well as developing risk analysis methodologies. The known reports about cybersecurity in the mining sector (e.g., [3]) define the most frequent motivation of the attackers (e.g., cyber espionage campaigns, classic thefts, etc.). However, the motivation of these attackers is usually presented in relation to the automation level of mines, goal/business impact, mode of attack, and attackers’ profile/model (e.g., hacktivists, competitors, cybercriminal syndicates, employees, terrorists). The attackers’ profile/model, in turn, also depends on the attackers’ skills/knowledge or location (see [55]). In another work [56], the cyber attackers are characterized by taking into account, among others, their motivation, technique used, or types of targets.

Taking into account the presented above conditions/dependencies, as well as the authors’ expert knowledge on the operations of mining companies, the authors concluded that the practical approach for cyberattack risk analysis in the mining industry should be based on Kaplan and Garrick’s model, which was defined as follows [9]:

(1) $$R=\left\{S_{i},P_{i},C_{i}\right\},\;i=1,2,\ldots ,N$$

where:

*R*—risk;
{}—must be interpreted as a “set of”;
*S*—a scenario (undesirable event) description;
*P*—the probability of a scenario;
*C*—the measure of consequences caused by a scenario;
*N*—the number of possible scenarios.

Moreover, the main assumptions of the classical approach to risk assessment (described, among others, in the ISO 31000 standard [11]) are also satisfied.

**Figure 1:**
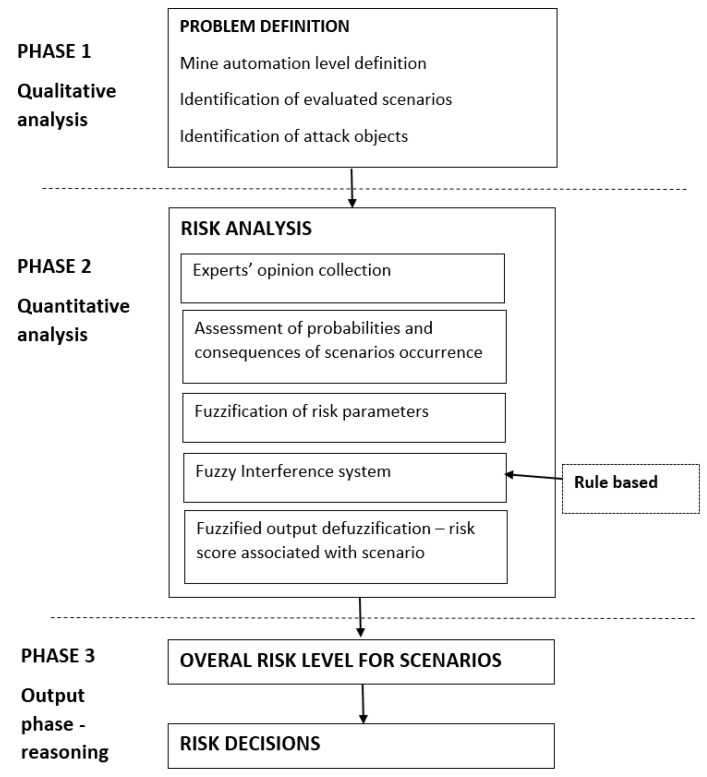
Risk analysis procedure in a mining company.

Following this, the proposed approach adopted in this study consists of three main phases. The first step is based on qualitative analysis implementation. During this phase, the identification of mine automation level and the definition of evaluated scenarios and attack objects are performed. The second phase includes quantitative risk analysis performance. At this stage, the collection of experts’ opinions about the probability and consequences of the occurrence of a given scenario, as well as risk quantification using fuzzy logic, are performed. The last phase—an output phase—provides risk level definition and reasoning on the level of risk obtained. Figure 1 represents the graphical view of the proposed complete methodology followed.

The first three steps of the analysis are used to identify the analyzed scenarios and determine the system’s vulnerability to the existing threat from a cyber-attack. The higher the automation of processes in the mine, the greater the possibility of achieving the spectacular effects of cyber-attacks. This causes an increase in the level of automation of mining processes, which increases their exposure to such undesired events. Thus, the adopted approach indirectly takes into account the motivation and profile of the attacker:

- Type of scenarios and automation type may be related to the attacker’s skills/knowledge;
- The attacker’s defined targets may be related to motivation/intention.

The next two steps of the procedure focus on estimating the parameters concerning the probability of a given scenario and its effects. Cyber-attacks are not highly repeatable events. Therefore, the mining company does not have historical data or experience that would allow the analysts to estimate both parameters’ actual level for a given object. Therefore, in order to determine the size of potential impacts and the frequency of occurrence of a given scenario, sectoral market analyses (allowing the determination of the specificity of cyberattacks occurring in the mining industry and potential impacts), as well as global market analyses (allowing the determination of the general trends of cyber-attacks) may be carried out. Due to the lack of possibility of using accurate statistical data, it was proposed in the described method to estimate both parameters by experts using the fuzzy logic concept. The analytical approach to determine these values is presented in Section 4.2. The last step in closing the risk analysis process is its evaluation. In this step, the overall scenario risk level is obtained. This acceptance level is individual and depends on the so-called “risk appetite” reported by the manager. Different working teams can observe a different risk acceptance level, which determines the managers’ further risk management activities.

### 4.1. Qualitative Analysis

The first phase begins with a description of the problem and the limits of the study.

Based on the literature review and the mentioned reports on registered cyber-attacks in the mining industry, the seven leading techniques used in these attacks have been distinguished. Each of the indicated attack techniques should constitute a separate scenario for an adverse event, which is subject to risk analysis. Table 1 presents the essential characteristics of each of the analyzed scenarios.

**Figure 2:**
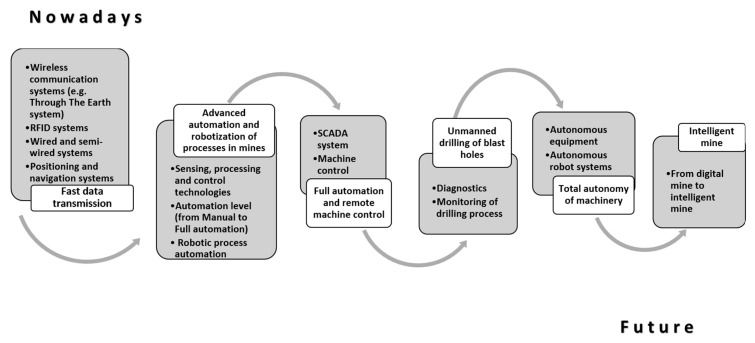
Process automation levels in a mining company (own contribution based on [57,58,59]).

An important aspect influencing the estimation of the other two elements describing the risks of a given scenario is the automation level in the analyzed company. In the mining sector, the literature [57] distinguishes six levels of mine automation, which are shown in Figure 2.

However, to develop a risk assessment method for the mining sector, three levels of mine automation have been proposed. These levels are described in Table 2.

The proposed method of analysis is dedicated to the mining sector. The conducted literature analyses allowed for the identification of three main areas (elements of the mining system) that may be the targets of the cyberattack. These are:

- PA1: attack on databases containing sensitive data and company know-how.
- PA2: attack on IT systems powering technical devices’ operation (e.g., machines, ventilation, air conditioning).
- PA3: attack on the company’s internal network, ICT devices used in the mine’s current operations.

**Figure 3:**
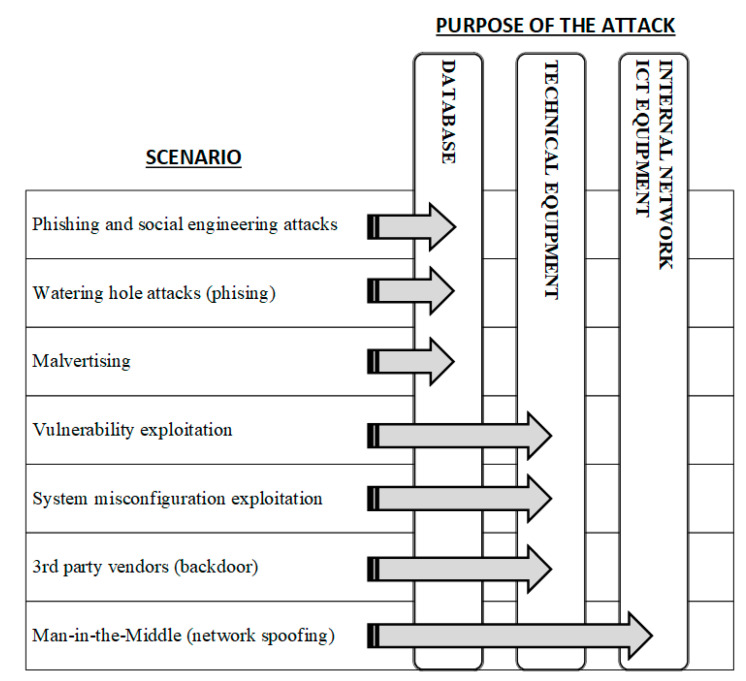
Assigning scenarios to attacked objects.

When formulating the risk assessment model, it has been noticed that individual attack techniques can be assigned to areas to which the attack is targeted. Therefore, the analyzed scenarios have been assigned to the object on which a given attack technique is directed. The results are presented in the form of Figure 3.

### 4.2. Qualitative Analysis: Assessment of Risk Score Associated with Given Scenarios

At the preliminary stage of this phase, the collection of experts’ opinions is performed. As a result, the experts provide their opinions for the values of probability and consequence for each identified scenario. The experts’ opinions on the defined risk parameters are collected using linguistic scales. The proper definition of linguistic variables is based on expert knowledge and depends on the industry type. The examples of the definition of linguistic variables for risk in the mining industry are given, e.g., in [48,49].

In the next step, they are modeled using fuzzy set theory.

The fuzzy set theory makes the comparison process more confident and increases the ability to explain the expert evaluation [60]. Therefore, the risk parameters of each scenario are treated as intuitionistic triangular fuzzy numbers (FN). The risk level is defined with the use of trapezoidal fuzzy numbers.

A triangular FN is presented by a triplet *A_z_* = (*a*, *b*, *c*), and its membership function is given by:

(2) $$\mu_{z}\left(x\right)=\{\begin{array}{c} 0\;for\;x<a\\ \begin{array}{c} \frac{x-a}{b-a}\;for\;a\leq x\leq b\\ \frac{c-x}{c-b}\;for\;b\leq x\leq c\\ 0\;for\;x>c \end{array} \end{array}$$

The FN parameters’ meaning is straightforward: *a* and *c* are the lower and upper bounds of fuzzy number *A_z_*, respectively, and *b* denotes the modal value of fuzzy number *A_z_*.

Following the previous notations, a trapezoidal FN is defined as *A_z_* = (*a*, *b*, *c, d*) and has its membership function given by:

(3) $$\mu_{z}\left(x\right)=\{\begin{array}{c} 0\;\;f<a\\ \frac{x-a}{b-a}\;for\;a\leq x\leq b\\ 1\;\;for\;b\leq x\leq c\\ \frac{d-x}{d-c}\;for\;c\leq x\leq d\\ 0\;for\;x>d \end{array}$$

Consequently, the linguistic variables with triangular and trapezoidal membership functions are presented in Table 3 and Table 4. The output variables for the risk score are given in Table 5.

Although the authors proposed triangular FN in this approach, there are few methods for assessing membership functions to fuzzy variables. For more information, see ref. [44].

Based on the experts’ opinions, there is a necessity to aggregate them to obtain the risk level. According to [61], the aggregation of experts’ opinions can be performed using the arithmetic mean aggregation operator. The mean aggregation operator, defined on fuzzy triangular numbers (*a*_1_, *b*_1_, *c*_1_), (*a*_2_, *b*_2_, *c*_2_)… (*a*_n_, *b*_n_, *c*_n_), delivers the result as (*x, y, z*) according to the formulae:

(4) $$\{\begin{array}{l} x=\frac{1}{n}\sum_{k=0}^{n}a_{k}\\ y=\frac{1}{n}\sum_{k=0}^{n}b_{k}\\ z=\frac{1}{n}\sum_{k=0}^{n}c_{k} \end{array}$$

The next steps of this phase are connected with risk quantification. This process is based on Mamdani fuzzy model use [62]. The Mamdani fuzzy interference mechanism is based on the compositional rule of inference proposed by Zadeh [41]. It is also one of the most commonly used fuzzy interference systems (see ref. [44] for more information).

**Figure 4:**
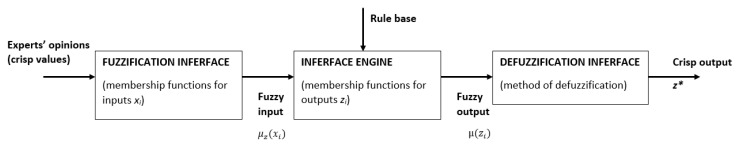
Stages of the use of fuzzy sets according to Mamdani fuzzy model.

The main components of the Mamdani fuzzy model are fuzzification, knowledge base, fuzzy interference system, and defuzzification [48]. Figure 4 represents the graphical view of the implemented Mamdani fuzzy model in the defined research area.

As was mentioned, the fuzzification process is based on the use of TFN. A triangular FN converts the linguistic scales in the range of 0–1 using its membership function.

The knowledgebase consists of the rule base and membership functions of inputs. The rule base includes a number of *IF-THEN* rules used to capture the imprecise modes of reasoning [52].

The fuzzy interference system (FIS) role is to map the fuzzy inputs and rule the outputs using a fuzzy set theory. Due to the Mamdani model use, the FIS is based on MIN and MAX operator implementation. The MIN operator is used for combination and implication operations. The MAX operator is used to aggregate the fuzzy outputs.

Finally, the defuzzification process is aimed at the conversion of the fuzzy output into crisp output. Thus, the centroid of area defuzzification method is used (see [63,64,65]). Following this, the output is estimated as [48]:

(5) $$Centroid\;of\;area,z^{*}=\frac{\int \mu_{A}\left(z\right)\cdot zdz}{\int \mu_{A}\left(z\right)dz}$$

where:

z*—the crisp value for the z output (defuzzified output);
$\mu_{A}\left(z\right)$—the aggregated output membership function;
*z*—universe of discourse.

As a result, the defuzzification process provides the crisp output value. This crisp output value is later implemented in the output phase to determine risk level.

### 4.3. Overall Scenario Risk Level Assessment

In the last phase, the defined scenarios’ risk level is determined, and risk decisions can be made accordingly.

The calculated risk indicators should form the basis for the management’s decisions regarding risk management activities. Actions are taken primarily in relation to scenarios for which the risk index assumes values that exceed the risk level that is acceptable to decision-makers. It should be noted that this level is not uniform, and, depending on the decision-makers, it may differ from mine to mine. It depends on the risk appetite of decision-makers, which determines their risk perception. Research indicates that this perception may depend on many factors, such as age, gender, character, and education [66].

For a scenario where the level of risk is not acceptable, managers should take the following actions:

- Risk reduction by (a) limiting the likelihood of the scenario occurrence. Taking protective/predictive actions, connected with introducing systems/procedures that secure external access to the company’s resources; (b) minimizing the effects of the scenario occurrence, building rapid response systems in relation to the occurrence of adverse events (resilience aspects).
- Risk transfer: transferring the effects of the occurrence of adverse events and their financial consequences to the insurer.
- Risk-retention: creating internal funds (financial reserves) to cover possible damages related to the occurrence of a given undesirable event.

Concerning the scenarios for which the risk indicator assumes values accepted by managers, decisions are usually made to accept this risk (not to take actions aimed at its reduction). At the same time, this risk should be periodically monitored so that it is possible to immediately take actions to reduce it to the acceptable level in the case of a change in its value.

## 5. Application of the Proposed Approach

To illustrate the proposed method applicability, the authors analyzed the seven defined scenarios concerning the type of mining company to be attacked. The analysis was based on the obtained survey research about cybersecurity in the business (see [67] for ref.). The possible consequences were analyzed according to the authors’ research undertaken in the frame of the SAFEME4MINE project and available literature (see [3,4,5,6,7,8,9,10,11,12,13,14,15,16,17,25,38,40] for ref.). Moreover, the authors based it on implementing methods such as brainstorming, accident or incident reports, and mine inspection reports. This phase gave the possibility of understanding what kind of consequences and frequency of scenarios can be defined and estimated. Additionally, the fuzzy rule-based risk assessment method is implemented using the fuzzy logic toolbox of MATLAB version R2020a.

The conducted analyses gave the possibility of obtaining some risk parameter estimations (see Table 6 and Table 7).

In the first step of constructing the proposed fuzzy model, the input parameters need to be fuzzified.

**Figure 5:**
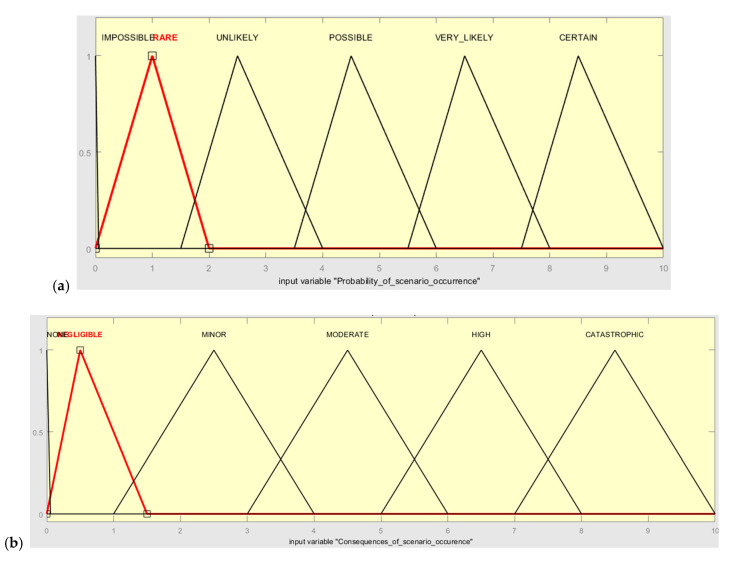

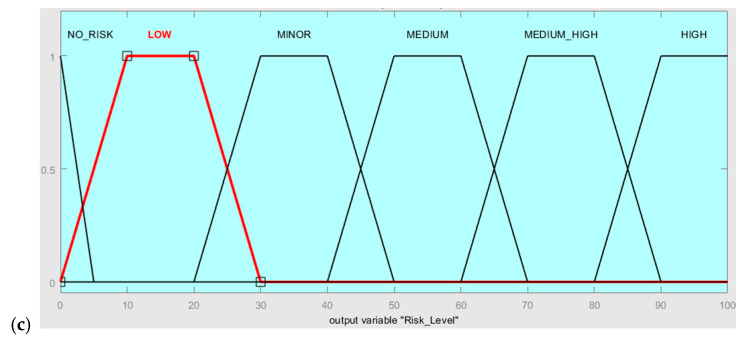
Membership function of (**a**) probability, (**b**) consequence, and (**c**) risk level.

Following this, the linguistic scores given by the experts are converted to corresponding fuzzy set numbers. In the developed Mamdani fuzzy model, probability and consequences are the two input variables and the risk level is the output variable. The triangular and trapezoidal FNs used in the presented case study to represent the linguistic scales of input and output parameters are shown in Figure 5 (according to Table 3, Table 4 and Table 5).

The final stage of constructing a fuzzy risk assessment model is to determine IF-THEN rules. According to the expert knowledge in underground safety analysis, 25 rules were proposed (presented in Table 8) and one additional, which defines the situation when there is no risk (rule 26). According to this, for example, rule one is defined as:

*IF probability is P1 and consequences are C1 THEN risk level is LOW.*

Rule 26 is defined as:

*If probability is impossible and there are no consequences, THEN the risk level is NO RISK.*

**Figure 6:**
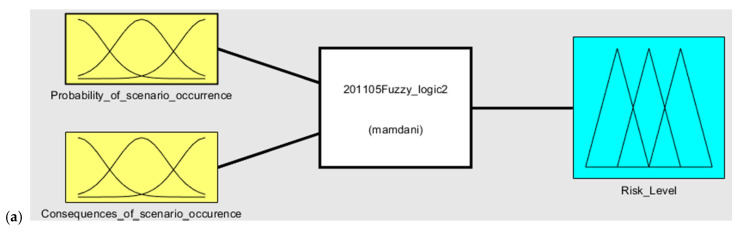

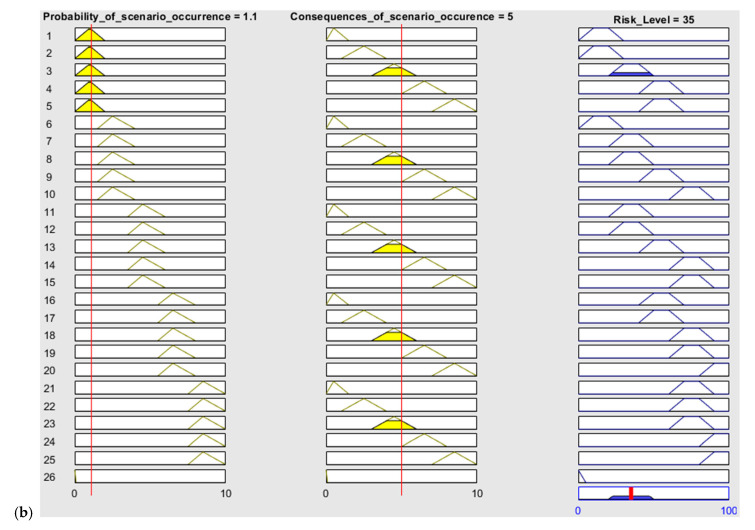
Sample (**a**) Mamdani model scheme and (**b**) rule base.

A fuzzy inference engine can aggregate and evaluate all of the fuzzy rules in the rule base, and the results of these rules are combined using the Mamdani algorithm (Figure 6). To obtain the final risk score from the constructed FIS, Equation (5) is used for the defuzzification of the fuzzy set resulting from the Mamdani algorithm.

Table 9, Table 10 and Table 11 present experts’ opinions for three investigated automation levels of mines. The consequence parameter is presented as an aggregated value of expert opinions. In Table 12 and Table 13, the obtained risk score is presented.

The results given in Table 12 are obtained by taking into account the assumptions that all decision rules have the same weights (the same importance). In the case where the weight of decision rules is not equal, the results are presented in Table 13. In such a situation, it is taken into account that the risk parameters have different importance and influence on the risk level. Bearing the above mentioned in mind, it is assumed that “catastrophic” consequences have more importance than “certain” probabilities in the risk assessment process. 

## 6. Results and Discussion

**Figure 7:**
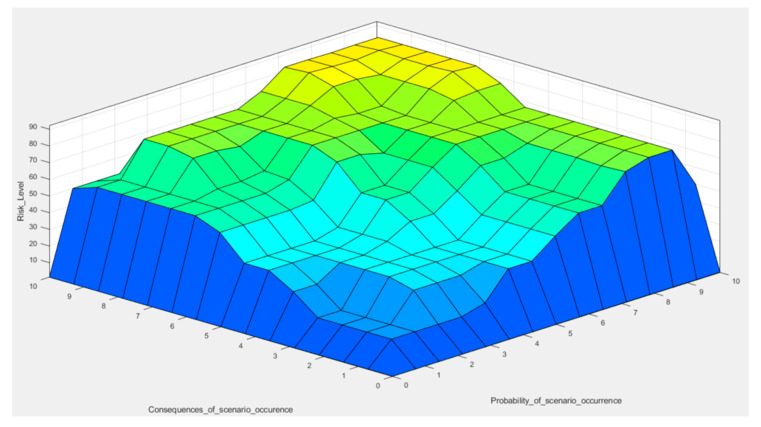
Surface view of the proposed fuzzy inference system (all rules are with weight = 1).

The proposed case study gives the possibility of analyzing how the developed risk decision method may be used to evaluate the risk level of cyber-attack in mines. The proposed method gives the possibility of employing linguistically exert knowledge and engineering judgment to make a more realistic evaluation in cybersecurity. The complete results of the proposed FIS for cyber-attack risk assessment are presented in Figure 7.

This 3D plot shows the resultant values of preliminary estimates: the probability of the occurrence of adverse events and their consequences being quantified based on expert knowledge. The consequence can be understood as an inability to maintain the operation, financial loss, failure, or reputational damage. The lowest, dark blue part of the plot represents the resultant level of risk caused by scenarios that occur relatively often, with insignificant damage potential. Maintaining the risk at this limited area is the safest from the risk management point of view. It should be mentioned that the uppermost corner, the yellow field, represents theoretically the most severe and certain risk (10/10 on the probability scale), which would affect the mine catastrophically. However, it presents only its hypothetical, highest level.

Knowing the current level of technological advancement (aforementioned level of automation), an entrepreneur can determine the chart’s area that describes existing risk levels related to mining operations. It may serve as an implicit indicator of the mitigation methods to be implemented. Assuming particular “risk appetite” of a decision-maker, retention, transfer, and reduction methods can be adjusted to the current and predicted situation to not exceed the light blue area of acceptable danger or to move from the upper zone to the lower, by, for example, reducing the probability of scenario occurrence. The plot provides a quantified basis for making managerial decisions regarding taking up active methods to reduce the risk or observing its level to determine when such actions should be implemented.

Based on the ranking of all of the scenarios obtained, a mitigation plan can be prepared accordingly so that preventive actions can be taken up for the riskiest hazard. As a result, the safety of a mine can be improved.

If the risk level is assessed at the HIGH level, it requires immediate actions aimed at reducing the likelihood of the occurrence of an attack and the absolute development of procedures to be followed in the event of an attack, which will minimize the consequences of its occurrence. Due to the catastrophic consequences, in this case, the mine should secure the necessary financial resources to cover possible losses (risk retention) as well as human and infrastructure resources to take over the necessary operational processes.

If the level of risk corresponds to the MEDIUM HIGH level, the mine’s management should also take preventive activities and minimize the consequences of the occurrence of a cyberattack. However, the time horizon for implementing the defined action may not concern the next weeks, as is the case with HIGH risk, but the period of 3 or 6 months. The implemented solutions should take into account both mine safety and an attempt to optimize their implementation costs. In this case, it is also reasonable to consider the possibility of insurance against the occurrence of selected scenarios.

In the case of the MEDIUM risk level, the company’s activities should be aimed primarily at minimizing the potential negative consequences of cyberattack occurrence. However, for the case of the defined scenarios, the development of a continuous monitoring system is essential. In this way, it will be possible to react quickly in the event of a change in the value of each of the analyzed risk parameters (both probability and consequences). The Mine 4.0 Continuous Monitoring System can rely on decision support applications that use real-time information. However, in the case of modern mines, a similar role may be played by the traditional system of monitoring indicators. However, then the system response time will be extended.

For LOW and MINOR risk levels, implementing proactive measures to lower the risk score may, in most cases, prove to be too costly. For this reason, a recommended action will be to periodically monitor the value of the risk indicator so that the system oversees the overall safety of the mine.

Additionally, a visible limitation of the analyzed application case is the assumption that the probability of the occurrence of individual scenarios remains at the same level, regardless of the level of mine automation. The assumption adopted in the analyzed case study results from the use of the research results on cyber-attacks prepared for the entire mining sector; hence, the lack of differentiation of this risk parameter concerning the analyzed variants of the mine automation level. In reality, however, the probability of a given scenario will differ in each of the analyzed variants. The higher the level of automation of the mine, the greater the “potential for external attacks”. The most spectacular effects of cyber-attacks appear in the case of Factory 4.0, so these objects are more often the target of attacks than traditional enterprises.

For this reason, the systems reporting data on cyberattacks and the effectiveness of countering them are of such importance for the needs of the prepared analyses. As a result, the mine will be able to base its analyses on current data on direct impacts on its own network and not rely on general industry data. This will increase the credibility of the results obtained and allow better management of the existing risk.

## 7. Conclusions

Cybersecurity in mines is one of the most important issues nowadays. Therefore, the mine’s risk assessment in the frame of Industry 4.0 solutions and automation level needs to be studied in detail. In this study, the authors’ research has been focused on the development of risk analysis methodology, which would take into account the specificity of the mining sector and the possibility of gathering data necessary for conducting the risk analyses in mines in relation to cybersecurity theory. The adopted approach was based on Kaplan and Garrick’s model [9] and is compatible with standard risk assessment methodology (according to ISO standard). Following the conducted literature research study and based on the direct interviews with operation managers in Polish mines, this approach was the most appropriate for the analyzed industry sector.

Moreover, the fuzzy logic structure allowed the experts to present the experts’ opinions in linguistic terms for the two defined risk parameters and to evaluate the risk level. The proposed case study shows the possibilities of using a given method in the decision-making process. However, the quantitative estimation of the risk parameters based on further experts’ opinions may be extended by using real data from various mines. The probability of scenario occurrence was estimated based on the cybersecurity reports available in the literature. The next step is to analyze a specific mine case, and according to its automation level and history of occurrence of cyber-attacks, this parameter may be estimated and implemented in the proposed risk assessment method. The same approach applies to the definition of consequences for the analyzed scenario. The assessment and experts’ opinions may vary according to the specific company and the mining industry experience.

In addition, the developed risk analysis method is based on expert knowledge and therefore, is burdened with a certain subjectivism resulting from the experience and knowledge of experts. As a result, the further direction of the conducted research is to strive for objectivity based on reliable quantitative data use. However, following the performed literature survey, the authors may state that there are currently no data available to allow such studies to be carried out for the entire mining sector. Therefore, the main future research directions aimed at extending the proposed method should be focused on analyzing the cybersecurity level of specific mines, which can represent the proposed levels of automation. Consequently, it will be possible to verify whether the risk results for individual representatives correspond to the results obtained, which are presented in this study based on the expert analyses. This requires extensive research, which would involve many mines with different process automation levels, but the results would be precious for the investigated industry sector.

To conclude, the proposed methodology could be used for cybersecurity risk assessment to be performed by mining management and safety officers. It may be useful for the risk prioritization process. Moreover, it gives preliminary information that can be useful for the development of risk mitigation methods as well as the selection of the most hazardous areas in the cybersecurity of the mines. Therefore, it provides essential information on the need to control the risk and to implement safety improvements.

The obtained results reveal the current cybersecurity status of the mine and are a valuable guide for mine authorities in facilities where automation-related actions are planned. Moreover, the proposed risk assessment method may be used in any type of mines, surface mining, and subsurface (underground) mining.

## Figures and Tables

**Table 1:** Description of the analyzed scenarios.

No. Scenario	Attack Technique	Description
S1	Phishing and social engineering attacks	Using crafted e-mails or effective social-engineering methods to gain confidential information
S2	Watering hole attacks (phishing)	An attack directed at a specific group, e.g., company employees of the relevant department, which leads to the impersonation of the attacker to the site, which the team uses in their daily work
S3	Malvertising	Injecting malicious or malware-laden advertisements into legitimate online advertising networks and webpages
S4	Vulnerability exploitation	Using an unaddressed system or software bugs to take control of them by using a dedicated program, often undetectable by the user or system operation
S5	System misconfiguration exploitation	Discovering the faults or flaws of unpatched yet software and exploits them to compromise the system
S6	3rd party vendors (backdoor)	Accessing the internal, corporate networks using back doors, embedded with specific functions or programmable implemented features in devices working in an internal company network
S7	Man-in-the-Middle (network spoofing)	An attack in which adequately prepared data packets are sent to impersonate another device on the network. An attacker appears between the user and the application, intercepting data packets, or impersonating one page. This can be done by, for example: accessing a router or making a prepared e-mail with a link to a website that looks like a real one and intercepting data transmission

**Table 2:** Characteristics of the mine automation levels.

Level	Name	Description
**L1**	**Modern mine**	A system, which enables operational and decisive support and/or remote aid. Locating mobile machinery and people is possible, but an only presence in certain zones can be identified to a reduced extent. Constant, real-time tracking is unavailable due to the lack of wireless communication infrastructure. Features characteristic for a modern mine: communication and data transmission network; advanced and widespread mechanization; partially automated processes; computer-aided maintenance; computer-aided works planning; cyclical analyses of risk and resources; constant output analysis. The human’s role is to operate the mobile machinery in person (one operator per machine needed) and supervise other machines’ functioning.
**L2**	**Real-time mine**	Particular, non-complex, repetitive tasks are performed using an automated system. A system can be described as ‘occasionally-autonomous’. Decisions about the transition from non-autonomous to the autonomous state have to be always confirmed by an operator. Limited autonomy is enabled due to the accurate monitoring of workplaces. Remote the machines’ operation allows to keep people away from dangerous mining fields and limit exposure to arduous working conditions. Operators/supervisors in control centers who are always alerted when an extraordinary situation occurs, taking control over or stop machines at any time. Features characteristic for a real-time mine: mine’s informatics web; widespread autonomous machinery, process-oriented production, non-human-maintenance production; remote control possible at all production steps; real-time risk, resources, and output analyses; data available in local control centers, ventilation-on-demand solutions. Human’s role changed for supervision and remote control of one or more machines simultaneously, planning and scheduling the works to be done by the autonomous machines.
**L3**	**Intelligent mine**	A system fully controls and partially supervises production processes, learning to base on the analyses of the consequences of its past actions. Activities can be performed entirely autonomously, even in variable characteristics of the work environment and changeable output demands. An instance of ‘intelligent’ interconnected processes in the mine can be an adaptive autonomous loading of the ore combined with an autonomous haulage system, both tuned up to the output from various working fields to obtain constant, satisfactory product quality demanded by the processing plant with no-human involved in the operation. Features characteristic for an intelligent mine: broad informatics web, connecting it with more mines to enable the production optimization; fully automated processes; automated supervisory systems; real-time tracking of all the machines; only autonomous and remotely controlled (in the most complex cases) mobile machinery; real-time, advanced: risk, resources and output analysis with related data available in a global control center. The human’s role is to analyze data, maintain the software and infrastructure, and almost 100% of the labor can be done remotely.

**Table 3:** Probability of scenario occurrence.

Rating Category	Description	Fuzzy Value
CERTAIN (P5)	Expected to occur regularly under normal circumstances	(7.5, 8.5, 10)
VERY LIKELY (P4)	Expected to occur at some time	(5.5, 6.5, 8)
POSSIBLE (P3)	May occur at some time	(3.5, 4.5, 6)
UNLIKELY(P2)	Not likely to occur in normal circumstances	(1.5, 2.5, 4)
RARE (P1)	Could happen but probably never will	(0, 1, 2)

**Table 4:** Consequences of scenario occurrence.

Rating Category	Description	Fuzzy Value
CATASTROPHIC (C5)	Multiple fatalities, catastrophic business loss	(7, 8.5, 10)
HIGH (C4)	Major injury, a single fatality, critical process loss, critical property damage	(5, 6.5, 48)
MODERATE (C3)	Reportable injury, moderate loss of process, limited property damage	(3, 4.5, 6)
MINOR (C2)	Non-reportable injury, minor loss of process, or slight property damage	(1, 2.5, 4)
NEGLIGIBLE (C1)	Minor injury, insignificant property or equipment damage	(0, 0.5, 1)

**Table 5:** Risk level.

Rating Category	Description	Fuzzy Value
HIGH (R5)	Consequences are catastrophic or high for a cyber-attack, which occurs undoubtedly or almost certainly in the future.	(80, 90, 100 100)
MEDIUM HIGH (R4)	An almost certain or certain event that occurs with minor consequences or the unlikely event that may have critical consequences	(60, 70, 80, 90)
MEDIUM (R3)	The probability of an event occurring is almost certain, and the consequences are minor or negligible, or the consequences are high, but the event is unlikely to occur	(40, 50, 60, 70)
MINOR (R2)	The occurrence of an event is possible with limited/not significant consequences	(20, 30, 40, 50)
LOW (R1)	The occurrence of the event is almost impossible. The consequences are insignificant	(0, 10, 20, 30)

**Table 6:** Probability of scenario occurrence.

Attack Technique	Occurrence Probability According to Cybersecurity Reports [%]
**S1**	72
**S2**	27
**S3**	27
**S4**	33
**S5**	9
**S6**	10
**S7**	5

**Table 7:** Consequences of scenario occurrence according to the targets of attack.

Attack Technique	Target of Attack	Consequences
**I—Modern Mine**		
**S1** **S2** **S3**	Databases (technical data, economic reports, personal data).	Financial loss related to data recovery (e.g., ransom), system updates (in order to reinforce uncovered weaknesses), and compensations for the employees whose data has been exposed to the attack; Loss of intellectual property, latent technology; Reputational damage.
**S4** **S5** **S6**	Mobile machinery (SCADA systems, onboard hardware related to the control of the machine); Control devices related to other machines and facilities in the mine (e.g., dewatering system, ventilation system).	Unplanned, manual inspections to be done; Increased wear and more frequent damages caused by the lack of information regarding malfunctioning; Obstructed performance analysis and optimization due to the lack of information from SCADA systems; Inability to adjust the energy supply to the actual demand; Limited access to workplaces due to ineffective water drainage; The limited ability of natural hazards’ evaluation.
**S7**	Communication and information networks.	Unauthorized access to network devices and loss of their configuration control; Interception of the data transmitted in an internal network (even if encrypted).
**II—Real-Time Mine**		
**S1** **S2** **S3**	Databases (technical data, economic reports, personal data)	The same as on the previous level – a more extensive scale.
**S4** **S5** **S6**	Mobile machinery (SCADA systems, onboard hardware related to the control of the machine); Control devices related to other machines and facilities in the mine (e.g., dewatering system, ventilation system); Autonomous machines.	As on the previous level, additionally: Machinery damage resulting from the continuation of work even if exceeding the permissible working parameters; Impossibility of local autonomous work due to malfunctioning of the machine control system - decrease in productivity and/or forecasting of the mining and weaning process; Reduction in the safety level resulting from the impossibility of locating workers and machines on the branches and in the ancestors; Need to switch to ‘traditional’ ventilation—increased costs.
**S7**	Communication and information networks.	The same as on the previous level—a more extensive scale.
**III—Intelligent Mine**		
**S1** **S2** **S3**	Databases (technical data, economic reports, personal data	The same as on the previous level—a more extensive scale.
**S4** **S5** **S6**	Autonomous machinery; Control devices related to other machines and facilities in the mine (e.g., dewatering system, ventilation system).	As on the previous level, additionally: Damage resulting from the loss of control over the operation of machines; Damage to machines as a result of working in inappropriate environmental conditions or being in the area of natural hazards; Stopping the production of entire departments or even the entire mine as a result of failure/damage to the main part of the vertical transport infrastructure
**S7**	Communication and information networks.	The same as on the previous level—a more extensive scale

**Table 8:** Risk decision matrix.

**P5**	MEDIUM HIGH	MEDIUM HIGH	MEDIUM HIGH	HIGH	HIGH
**P4**	MEDIUM	MEDIUM	MEDIUM HIGH	MEDIUM HIGH	HIGH
**P3**	MINOR	MINOR	MEDIUM	MEDIUM HIGH	MEDIUM HIGH
**P2**	LOW	MINOR	MINOR	MEDIUM	MEDIUM HIGH
**P1**	LOW	LOW	MINOR	MEDIUM	MEDIUM
	**C1**	**C2**	**C3**	**C4**	**C5**

**Table 9:** Expert opinions for all considered scenarios—modern mine I.

Scenario	P (Fuzzy)	P (Crisp)	C (Fuzzy)	C (Crisp)
**S1**	P4	6.67	(1, 2, 3.33)	2.11
**S2**	P2	2.67	(1.5, 2.5, 4)	2.67
**S3**	P2	2.67	(2.833, 3.833, 5.33)	4.00
**S4**	P3	4.67	(4.167, 5.167, 6.67)	5.33
**S5**	P1	1.00	(4.83, 5.83, 7.33)	6.00
**S6**	P1	1.00	(5.5, 6.5, 8)	6.67
**S7**	P1	1.00	(6.83, 7.83, 9.33)	8.00

**Table 10:** Expert opinions for all considered scenarios—real-time mine II.

Scenario	P (Fuzzy)	P (Crisp)	C (Fuzzy)	C (Crisp)
**S1**	P4	6.67	(4.83, 5.83, 7.33)	6.00
**S2**	P2	2.67	(4.83, 5.83, 7.33)	6.00
**S3**	P2	2.67	(5.5, 6.5, 8)	6.67
**S4**	P3	4.67	(6.167, 7.16, 8.67)	7.33
**S5**	P1	1.00	(6.83, 7.83, 9.33)	8.00
**S6**	P1	1.00	(6.83, 7.83, 9.33)	8.00
**S7**	P1	1.00	(7.5, 8.5, 10)	8.67

**Table 11:** Expert opinions for all considered scenarios—intelligent mine III.

Scenario	P (Fuzzy)	P (Crisp)	C (Fuzzy)	C (Crisp)
**S1**	P4	6.67	(4.167, 5.167, 6.67)	5.33
**S2**	P2	2.67	(5.5, 6.5, 8)	6.67
**S3**	P2	2.67	(6.167, 7.16, 8.67)	7.33
**S4**	P3	4.67	(6.83, 7.83, 9.33)	8.00
**S5**	P1	1.00	(6.83, 7.83, 9.33)	8.00
**S6**	P1	1.00	(7.5, 8.5, 10)	8.67
**S7**	P1	1.00	(7.5, 8.5, 10)	8.67

**Table 12:** Risk score for scenarios vs. mine automation level (all rules are with weight = 1).

Scenario	Risk Score (Modern Mine)	Risk Score (Real-Time Mine)	Risk Score (Intelligent Mine)
**S1**	55	75	75
**S2**	35	55	55
**S3**	35	55	61.7
**S4**	61.7	75	75
**S5**	55	55	55
**S6**	55	55	55
**S7**	55	55	55

**Table 13:** Risk score for scenarios vs. mine automation level (added weights to the rules).

Scenario	Risk Score (Modern Mine)	Risk Score (Real-Time Mine)	Risk Score (Intelligent Mine)
**S1**	55	75	75
**S2**	35	55	55
**S3**	35	55	61.4
**S4**	58.3	75	75
**S5**	55	55	55
**S6**	55	55	55
**S7**	55	55	55
